# Breastfeeding Perceptions and Decisions among Hispanic Participants in the Special Supplemental Nutrition Program for Women, Infants, and Children: A Qualitative Study

**DOI:** 10.3390/nu16111565

**Published:** 2024-05-22

**Authors:** Emily Fisher, Priyanka Patel, Kathryn G. Wouk, Bidusha Neupane, Futun Alkhalifah, Marilyn M. Bartholmae, Chuanyi Tang, Qi Zhang

**Affiliations:** 1 School of Community and Environmental Health, Old Dominion University, Norfolk, VA 23529, USA; efish008@odu.edu (E.F.); ppate008@odu.edu (P.P.); bneup001@odu.edu (B.N.); falkh003@odu.edu (F.A.); barthomm@evms.edu (M.M.B.); 2 Pacific Institute for Research and Evaluation, Chapel Hill, NC 27514, USA; kwouk@pire.org; 3 Department of Psychiatry and Behavioral Health, Eastern Virginia Medical School, Norfolk, VA 23510, USA; 4 Department of Marketing, Old Dominion University, Norfolk, VA 23529, USA; ctang@odu.edu

**Keywords:** WIC, breastfeeding, Hispanic, infant feeding decision

## Abstract

The Special Supplemental Nutrition Program for Women, Infants, and Children (WIC) is a nutrition assistance program in the U.S. WIC served 2.5 million eligible Hispanic women, infants, and children under the age of five in 2021, which is WIC’s largest racial/ethnic group. However, limited research has been conducted to understand Hispanic WIC participants’ perceptions of WIC breastfeeding recommendations and their breastfeeding decisions. For this qualitative study, we interviewed 18 of these pregnant and postpartum WIC participants on their experiences and decision-making processes related to breastfeeding. Hispanic cultures and home country norms were identified as prominent influences on breastfeeding decisions, along with perceptions of WIC’s breastfeeding support. These results can help the WIC program to refine its breastfeeding education to better meet the needs of Hispanic participants.

## 1. Introduction

Breastfeeding is associated with numerous maternal and infant health benefits [1]. As a result of these well-established benefits, consensus guidelines from major medical and public health organizations recommend six months of exclusive breastfeeding, with continued breastfeeding through the first year “or longer as mutually desired by the woman and her infant” [2,3]. Breastfeeding promotion and support are integral components of the Special Supplemental Nutrition Program for Women, Infants, and Children (WIC), which serves nearly half of all infants born in the U.S. [4]. However, providing free formula benefits to WIC participants may undermine WIC’s breastfeeding promotion and contribute to lower breastfeeding rates among WIC participants compared with eligible or ineligible non-participants [5,6].

Prior research suggests that WIC participants’ prenatal perception of WIC’s breastfeeding recommendations significantly predicts multiple breastfeeding outcomes in the first year after birth. Using a national longitudinal sample of 1594 pregnant participants in the WIC Infant and Toddler Feeding Practices Study-2 (WIC ITFPS-2), researchers found that if pregnant WIC participants perceived that WIC recommends breastfeeding only, they had a significantly longer breastfeeding duration and exclusivity than peers who perceived that WIC recommends formula only or both breastfeeding and formula equally [7,8]. In subsequent qualitative research with WIC participants, key factors were identified that influenced how participants formed their perception of WIC’s infant feeding recommendations, including the mother’s social network and healthcare providers, the WIC program’s media and breastfeeding promotion, and the program’s long history as a distributor of free formula [8]. Understanding these mechanisms can inform the adaptation and targeting of prenatal messaging to improve breastfeeding in WIC populations. 

While these findings contribute to a growing field of intervention science to improve breastfeeding in WIC populations, the research thus far has been limited to the experience of English-speaking WIC participants. The Hispanic population is the largest racial/ethnic minority in the U.S., comprising 19.1% of the population [9]. Since 2017, the Hispanic population has surpassed the non-Hispanic White population as the largest racial/ethnic group eligible for and participating in WIC [4]. Therefore, understanding Hispanic WIC participants’ perceptions of WIC’s infant feeding recommendations and their breastfeeding decision process is critical to shaping WIC breastfeeding education and supporting policies for Hispanic participants.

While many Hispanic WIC participants have been excluded from research due to language barriers, the WIC ITFPS-2 data have shown that Hispanic WIC participants were more likely than non-Hispanic Black and White participants to intend to breastfeed, and yet they are less likely to meet their breastfeeding goals [10]. One qualitative study exploring infant feeding decision making in a sample of low-income Latina mothers found a perception that formula was healthier than breastfeeding because of its provision by public health programs like WIC [11]. Other research shows that low-income Hispanic participants describe WIC as a prominent source of information that influences their infant feeding decisions [12]. It is, therefore, noteworthy that Hispanic mothers are more likely than non-Hispanic mothers to mix breastfeeding with formula supplementation [13], a practice associated with shorter breastfeeding duration and increased risk of childhood obesity [14]. One possible reason for this practice may be that Hispanic populations receive less breastfeeding support than non-Hispanic populations, as studies have documented stereotyping by healthcare providers who assume that “Hispanics do *las dos cosas* (both infant formula feeding and breastfeeding)” [15,16]. Hispanic mothers who have immigrated to the U.S. may also perceive pressure to adopt formula feeding as they acculturate to the U.S. context, despite cultural norms in their countries of origin to breastfeed [17]. 

More research is needed to understand how Hispanic WIC participants form their prenatal perceptions of WIC’s recommendations and decisions on breastfeeding in the context of experiences, cultural norms, and socioeconomic challenges. In this qualitative study, we aimed to build upon prior research by conducting in-depth interviews with Hispanic WIC participants to understand how they form their perceptions about the program’s breastfeeding recommendations and to identify mechanisms that might influence their breastfeeding decisions. 

## 2. Materials and Methods

Given that little is known about Hispanic WIC participants’ breastfeeding decision making, a qualitative research design was appropriate to obtain a deep understanding of the factors that influence their breastfeeding practices. We conducted in-depth interviews with Hispanic WIC participants to understand how they formed their perceptions of WIC infant feeding recommendations and what factors contributed to their decisions on breastfeeding. The interview data were then transcribed, coded, and analyzed.

### 2.1. Participant Recruitment

The study received ethical approval from the Institutional Review Board (IRB) at Old Dominion University, ensuring compliance with all the necessary ethical standards. To recruit participants, we collaborated with Nevada’s WIC state agency to identify Hispanic pregnant and postpartum participants. Our recruitment strategy used the WICShopper app, the cell phone app for Nevada WIC participants, which directed interested participants to a Qualtrics survey in Spanish for informed consent and eligibility screening. The inclusion criteria were being Hispanic (self-reported) and being first-time pregnant (pregnant participants) or having given birth for the first time within the prior six months (postpartum participants). If the participant was eligible, the survey collected the participant’s socio-demographics, including pregnancy status, age, marital status, educational level, employment status, health insurance, language spoken at home, urban/rural status, and residence in the U.S. Eligible participants provided their phone numbers so researchers could contact them and conduct in-depth interviews. Monetary compensation was provided to each participant who completed the eligibility survey.

### 2.2. In-Depth Interviews

Two bilingual (Spanish–English) researchers called and interviewed the enrolled participants in Spanish using a semi-structured interview guide. The interview guide, developed based on a comprehensive literature review and expert input, aimed to understand Hispanic WIC participants’ perceptions of WIC’s breastfeeding recommendations, the cultural and social factors influencing these perceptions, their prenatal infant feeding intentions, and their actual infant feeding practices (if postpartum). Each interview proceeded only after obtaining the participant’s informed consent and began with broad and open-ended questions, followed by further probing when necessary. 

For example, pregnant mothers were asked, “How do you plan to feed your new baby in the first few weeks?” and “What made you decide how to feed your new baby?”. These questions were asked to understand the initial intentions, influences, and education behind their feeding choices. For the postpartum mothers, key questions included “How did you feed the baby when you were in the hospital and at home?” and “How did you decide to feed your baby the way you’re feeding him?” to capture and understand their feeding practices at different points in time and the factors shaping these behaviors. Additionally, questions such as “Could you tell me about your experience using WIC?” and “Do you know if WIC has any recommendations about how to feed the newborn baby?” were used to assess participants’ experiences with WIC and to understand perceptions of educational information provided to mothers by WIC staff. To further explore the cultural influence on infant feeding decisions and practices, key questions like “How are babies fed in your Hispanic/Latina culture?” and “How has your Hispanic culture influenced your decision to feed your baby?” and “What is your view of Hispanic culture and practices regarding baby feeding?” were asked to obtain additional insight into factors that affect infant feeding decisions.

Additional interviews were conducted until the point of theoretical saturation was reached [18]. The interviews lasted for approximately 45 min to one hour. Each phone interviewee was given additional monetary incentives upon completing their interviews.

### 2.3. Qualitative Analyses

Once the researchers had conducted and recorded the interviews, the audio files were loaded into the Sonix platform for transcribing. Two Spanish–English bilingual researchers reviewed the Spanish and English transcripts for accuracy. Following the procedure recommended by the literature [19,20], the transcribed data were coded and analyzed. Specifically, the coding was conducted in three stages: open, axial, and selective [20]. In open coding, we identified a broad range of codes and themes such as family influence on breastfeeding, the early introduction of supplement feeding, home country breastfeeding norms, and changes in their views of formula after moving to the U.S.A. In the axial coding, we further refined the themes and categories and explored the relationships among the categories. For example, we found that the above four themes were connected and categorized them into a broader category of Hispanic culture. We also examined the relationships between this category and other categories. In the selective coding stage, we developed a theoretical framework to explain Hispanic WIC participants’ breastfeeding decisions. This framework identified three major contributors: Hispanic participants’ breastfeeding preferences, the importance of Hispanic culture, and Hispanic participants’ perceptions regarding WIC’s breastfeeding recommendation. To ensure the reliability of the coding, two researchers coded the data independently. The research team met regularly to discuss the findings and themes. When a coding disagreement occurred, the whole team was involved until the two coders reached an agreement. NVivo 12 was used in coding.

## 3. Results

Several major themes emerged in our data analyses. We first summarize the characteristics of our participants and then discuss the major themes below, including Hispanic WIC participants’ breastfeeding preference, the important role of Hispanic culture, and Hispanic participants’ perceptions of WIC’s breastfeeding recommendations.

### 3.1. Participant Characteristics

Of those who completed all the screening questions on the eligibility survey, 68 participants were identified as eligible. Out of these 68, 18 Hispanic participants agreed to participate in an interview. Seven participants were pregnant at the time of the interview, and eleven reported having delivered a child within the last six months. 

Table 1 reports the socio-demographics of the participants. There were no participants aged 40 or older. Concerning marital status, half of the participants were single, divorced, separated, or widowed. The educational accomplishments of the participants were diverse, with the largest proportion having graduated high school or obtained a GED. The participants’ employment status varied, with almost 90% being housewives, part-time workers, or other employees. The majority of the participants had some form of medical insurance. Although all participants preferred speaking Spanish in the interview, at home, approximately 40% of them spoke English only or both Spanish and English. Most participants resided in urban areas. The length of residency in the U.S. was heterogeneous. 

We summarized the themes into three parts: Hispanic participants’ infant feeding preferences, the impact of Hispanic culture, and their perception of WIC’s breastfeeding recommendations.

### 3.2. Hispanic Participants’ Infant Feeding Preference

#### 3.2.1. Hispanic Participants Have a Strong Preference for Breastfeeding

Table 2a (pregnant participants) and Table 2b (postpartum participants) report the descriptive results of Hispanic WIC participants’ breastfeeding planning, decision, and practice processes. In general, pregnant Hispanic participants expressed a strong preference for breastfeeding over formula feeding, citing the nutritional benefits and encouragement to breastfeed from both personal and professional networks. Five of the seven pregnant participants were in their second trimester. One was in her first trimester (participant #68, i.e., P68), and another one was in her third trimester (P40). They planned to breastfeed for 3 to 12 months. One participant who was in her first trimester was not aware of alternative feeding practices to breastfeeding.

*“Well, it’s [breast milk] more beneficial for the baby than formula… Breast milk is already something that is intended for the baby.”*

(P40)

*“Well, I’ve always seen that most of my family members have breastfed, and I’ve always heard the benefits.”*

(P56)

#### 3.2.2. Hispanic Participants Treat Formula as the Backup

All six pregnant participants reported formula as the backup for no or low milk supply (four participants) or job-related reasons (two participants). Almost all the participants acknowledged the difficulties associated with breastfeeding, mentioning how painful breastfeeding can be for participants and feeling uncertain about how much breastmilk they will produce or whether infants would learn to breastfeed. Hispanic participants reported that formula had both drawbacks and benefits, such as concerns about formula related to its inability to help build the infant’s immune system and its nature as a processed substitute for human milk. Yet, they acknowledged the necessity of formula as a backup option when common breastfeeding issues arise or when they must return to work.

*“Well, from what I’ve seen is that they eat every…every few hours. So I’ve been preparing for what this will be like…what breastfeeding is. But I still have my doubts because I do not know how, how my baby will react when breastfeeding.”*

(P17)

*“Well, it’s bad to give baby formula, he’s not going to have good defenses and things like that.”*

(P26)

*“I fed her with a formula for the first few days, and I extracted breast milk, and the nurses gave the breast milk to the baby… since I didn’t produce much breast milk, they gave her formula too.”*

(P1)

#### 3.2.3. Most Hispanic Participants Changed to Mixed Feeding When in the Hospital

Nine out of eleven postpartum participants planned breastfeeding during pregnancy, one planned mixed feeding (P1), and one was unsure about her feeding plan (P89). Both P1 and P89 practiced mixed feeding in the hospital. However, five of the nine participants who originally planned breastfeeding changed to mixed feeding in the hospital and kept the practice after discharge. Only three kept their original breastfeeding plans until the interview. One participant (P76) changed her practice to mixed feeding after five months of exclusive breastfeeding. The main reasons for change were participants’ low breast milk supply and infants’ high bilirubin levels.

*“What some mothers say that it is very tiring, that they have tried it (breastfeeding) but that for their health they end up doing mixed breastfeeding.”*

(P68)

*“Well, it depends on my body, if I have milk as soon as baby is born, I will breastfeed. But if not, then I will use the formula, but preferably breast milk as long as I can. As long as I have milk.”*

(P40)

In summary, most Hispanic participants planned breastfeeding during pregnancy and used formula feeding as a backup if breastfeeding was not successful. However, most postpartum participants changed to mixed feeding during their stays in the hospital. In addition, notably, participants did not mention any alternative support or resources to support breastfeeding through these experiences, such as seeing a lactation consultant, utilizing a breast pump, or accessing donor milk. This omission suggests a lack of knowledge regarding the various resources available to address breastfeeding challenges.

### 3.3. The Importance of Hispanic Culture

The interview data consistently show that the participants valued their Hispanic culture, and components of Hispanic culture, such as family and infant feeding norms, played an important role in Hispanic participants’ infant-feeding decisions. 

#### 3.3.1. Family Influence Is Significant for Hispanic WIC Participants

Pregnant participants recognized the value of consulting those with the lived experience of parenthood and infant feeding, inferring that they could benefit from the lived experience of maternal figures in their family and community. Postpartum participants also acknowledged that familial perspectives and experiences were influential in shaping their infant feeding practices.

*“Ah, well the help of the moms, and the family. Of those who have experience.”*

(P31)

*“Well, the thing is, they’ve already had better experiences, so they know what to expect and I don’t as a first-time mom. So I know if I follow their advice, I know I will do the right thing.”*

(P100)

*“Well, since they already have experience, yes, it is important to pay some attention to their opinions based on what they went through and their experiences.”*

(P17)

#### 3.3.2. Early Introduction of Supplemental Feeding in Hispanic Culture Contributes to Formula Adoption in the U.S.

Notably, the WIC participants expounded on how their Hispanic culture frequently encouraged the early introduction of supplemental foods for infants while also encouraging continued breastfeeding. The reasons given for adopting early supplemental infant feeding centered around wanting their infant to gain weight and sleep well, the need to build the infant’s stomach, using accessible alternatives to the formula in their countries of origin, and helping remediate other infant feeding issues. For example, solid foods like pumpkin porridge and “Mazamorras” or corn starch mixed with water should be introduced as early as four months of an infant’s age. However, several participants expressed concerns about following these cultural supplemental feeding practices, citing illogical reasoning and a lack of information as reasons for these practices.

*“They start with a little chamomile water, apple, lettuce, I don’t know what. After two months you can give them soups… and they have to be given what we call in quotes the ‘mazamorras,’ which are the corn starch that supplements formula milk. Lots of oatmeal.”*

(P45)

*“Ah, well. That’s very different from what I practice, because the babies there… yes, yes, people, as I mentioned, usually add pureed food or flour, the baby cereals, in the milk and for me that has always seemed absurd.”*

(P56)

*“Because like I said, sometimes they use things like to help the baby get chubbier or to cleanse the stomach I’ve heard too.”*

(P57)

#### 3.3.3. Home Country Infant Feeding Norms Focus on Breastfeeding Instead of Formula Feeding

Hispanic participants born outside of the U.S. consistently reported that their home country norms placed a substantial emphasis on breastfeeding as the preferred method for infant feeding. Both pregnant and postpartum participants reported that their home country norms did not respond favorably to formula use. Participants cited reasons for these views, such as the fact that no safe formula is available, the high cost, and the lack of health benefits to the baby.

*“Well in Cuba… children, if it is not with breastfeeding, there is almost no formula.”*

(P53)

*“More than anything, I know that in my country it is a lot… everyone, 90% of people are breastfeeding, and moms breastfeed.”*

(P76)

*“I know that the formula is very expensive. It is very expensive, and it does not offer as much.”*

(P76)

*“Well, it’s not good to give him that because the child is going to be very sickly. So it’s going to cause more harm if you feed the baby formula. They see it like that, at least where I am from.”*

(P26)

#### 3.3.4. Hispanic WIC Participants’ View of the Formula Changed after Moving to the U.S.

When asked about distinctions in infant feeding practices between Hispanic and US cultures, participants described cultural differences centering around access to services such as maternity leave, medical professionals, and supplemental feeding methods like formula instead of solid foods. Participants often reported poverty in their home countries as a critical factor leading women to adopt different infant feeding practices outside of breastfeeding or formula feeding. They described the complementary use of other liquids and solid foods alongside breastfeeding because the formula was too expensive or of poor quality. Additionally, participants cited their adaptation to American culture, noting that they had changed their view of formula after immigrating to America and perceived it as having more benefits associated with it.

*“Like with WIC. Or maybe with the doctors or with people, everything is more accessible here than in Latin American countries.”*

(P100)

*“I thought that the formula wasn’t so good, that it wasn’t the healthiest. But now I say it’s a blessing to have it because it’s a help.”*

(P76)

In summary, Hispanic culture and home country norms had a significant impact on Hispanic participants’ breastfeeding planning, decisions, and practices.

### 3.4. Hispanic WIC Participants’ Perceptions Regarding WIC’s Breastfeeding Recommendations

Previous studies [7,8] suggest that WIC participants perceived that WIC recommends formula only or both breastfeeding and formula equally, which may influence WIC participants’ infant feeding decisions. The Hispanic participants in our current study also mentioned that their perceptions of WIC breastfeeding recommendations influenced their infant feeding decisions. Thus, we investigated the Hispanic WIC participants’ perceptions of WIC breastfeeding recommendations, what factors contributed to the perceptions, and how WIC influenced Hispanic WIC participants’ infant feeding decisions. 

#### 3.4.1. Most Hispanic WIC Participants Believed WIC Recommends Breastfeeding, Although Their Perceptions Were Mixed

Most postpartum Hispanic participants and all pregnant participants indicated that they believed WIC recommends breastfeeding as a best practice for infant feeding. Two postpartum participants indicated that they believed WIC supports each participant’s infant feeding choice, whether breastfeeding, formula feeding, or combining the two. One postpartum participant reported that WIC recommended feeding the infant nutritious food, deducing that this meant that if she decided to breastfeed, she, as the person making the breastmilk, needed to be eating healthy foods as well. Additionally, another postpartum participant believed that WIC recommends formula because WIC staff had educated her on how to prepare it.

*“They are more supportive of breastfeeding.”*

(P26)

*“They only tell you about the things that you as a parent, that is, as a participant, choose between the two… that you as a mother choose.”*

(P56)

*“Obviously, breastfeeding requires that I first eat very well and also maintain a healthy life with respect to drinking a lot of water and avoiding saturated things… Good to avoid certain fats, increase vegetables and fruits.”*

(P75)

*“They told me how I had to prepare the formula, how to hold the baby, and some warning signs about the baby. Like when she’s choking and so on… I imagine that since I’m a first-time mom, it helps a lot because you don’t know for sure how to do things.”*

(P1)

#### 3.4.2. Hispanic WIC Participants’ Perceptions Mainly Come from WIC Education and Their Interactions with WIC Staff

Factors that influence Hispanic WIC participants’ perceptions of WIC’s infant feeding recommendations include educational and relational influences. Educational influences include both print and virtual media. Hispanic WIC participants specifically recalled receiving physical materials such as books, flyers, and brochures. Participants also reported visiting the WIC website and engaging with the WIC app to enhance their understanding of recommended infant feeding practices. Additionally, several participants indicated that the WIC nutrition classes provided them with infant feeding information. Participants also reported that relationships with WIC staff cultivated either through in-person communication, phone calls, or virtual meetings influenced their perceptions of WIC’s infant feeding recommendations.

*“They would call me and invited me to take some classes through the mail. I registered and I found it very interesting.”*

(P76)

*“Well, WIC calls me. They do video calls.”*

(P7)

*“They told me directly in the office.”*

(P31)

#### 3.4.3. Spanish Communication and Support from WIC Staff Helped to Shape a Positive View for Hispanic Participants

When asked if WIC supported their cultural infant feeding decisions and practices, participants consistently reported culturally appropriate communication in WIC offices, both with staff and the materials provided in Spanish. Hispanic participants were pleased with the ability of staff to speak their language and have translators available to assist with sensitive conversations, if needed. They also stated that the Spanish materials provided to them helped inform their choices around nutrition for themselves and their infants.

*“Yes, sure. I thought, I thought they [WIC staff] wouldn’t speak Spanish, because I don’t speak English. But it wasn’t very… they were very cordial. They treated me very well from the beginning and until now that I have gone to the appointments.”*

(P17)

*“Yes, yes, yes, absolutely. Both in person and in the app. Because in the app, eh well I have looked at tips or advice for… for recipes for babies or for what is good. And the books, too, which sometimes they give you to help you make the right choices.”*

(P100)

*“Yes, of course. They have always been very open about it. I have not felt discriminated against or complicated by my culture or my language, no.”*

(P26)

The culturally appropriate services provided to Spanish-speaking WIC participants were described as reassuring and helpful. Participants reported that nutritional information and financial assistance were crucial in supporting their infant feeding decisions. Program accessibility was also mentioned regarding the usefulness of the WIC app and the proximate location of WIC offices. Overall, participants reported feeling comfortable and supported by the services WIC staff provided. Participants recounted that WIC staff remained calm, were thorough in their responses, and reassured participants to build confidence.

*“Yes, because they are calm. They don’t try to scare first-time moms. They explain things to you.”*

(P31)

*“So I repeat, WIC was a great help because I did not know that after a miscarriage it is like a recovery, just like childbirth. So, it was like not only the foods, but also the counselors, because they called me, asked me how I felt, checking on my mood. And well, all that.”*

(P7)

*“Yes, sure. They have been very supportive because for a while, no, I didn’t have many resources to… to have everything at hand. Like economically. And the truth is, WIC has helped me a lot in the form of all the food they give me from the WIC.”*

(P89)

## 4. Discussion

Our findings suggest that Hispanic cultures play an important role in Hispanic WIC participants’ infant feeding decisions and practices. We found that most of our Hispanic participants plan to breastfeed, which is largely because Hispanic cultural norms traditionally prioritize breastfeeding. The study participants shared that their Hispanic cultural norms traditionally emphasize the importance of breastfeeding due to its recognized health benefits for both the infant and the mother. This cultural norm is strongly reinforced and encouraged by maternal figures, community support, and social media promoting breastfeeding as the preferred method of infant feeding. Such practices are not merely nutritional decisions but are integral to cultural heritage and are passed down through generations. This aligns with the findings by Galvez et al. (2018), who highlight the critical role of family structure and maternal influence in breastfeeding practices among Hispanic populations, reinforcing the notion that breastfeeding is not only a nutritional choice but also a cultural legacy passed down through generations [21]. Carlin et al. (2019) underscore the significance of community-based support systems in promoting breastfeeding, indicating that such networks can also greatly influence a mother’s decision to breastfeed [22]. Finally, Skelton et al. (2018) discuss the impact of digital platforms in circulating health-related information, noting how targeted social media campaigns can effectively encourage breastfeeding practices among Hispanic women [23]. Together, these studies underscore the multifaceted support systems that reinforce breastfeeding as a preferred and culturally significant practice within the Hispanic community.

We also found that most participants switched to mixed breast and formula feeding when encountering challenges in breastfeeding, which may be attributed to the culture of the early introduction of alternative foods. This finding aligns with the literature, which indicates a common practice among Hispanic mothers to introduce early supplementary feeding, reflecting deep-rooted cultural norms and socioeconomic factors [24]. The preference for early solid foods, as shared by participants, is partly due to the inaccessibility of formula in participants’ countries of origin. It aims to prepare infants for solid meals and build their stomachs while satisfying their hunger. Cartagena et al. (2014) further suggest such practices driven by the belief in the health benefits of chubbier infants may contribute to early mixed feeding and overfeeding tendencies [25]. The practice of introducing solids in the early months, which is deeply rooted in cultural traditions, undergoes a significant shift upon migration to the U.S., where the early introduction of solid foods alongside breastfeeding is often perceived negatively and discouraged by healthcare providers and WIC staff, as it contributes to reduced breastfeeding duration [26]. In contrast, healthcare professionals and WIC staff tend to recommend formula as an alternative in the face of breastfeeding challenges. Additionally, infant formula companies’ marketing strategies significantly shape these perceptions by aggressively advertising formula as a safe alternative [27,28].

The study further reveals participants’ perceptions surrounding the early introduction of formula as a backup measure for anticipated challenges such as insufficient milk production, infant satiety, latching issues, and the pain associated with breastfeeding. Moreover, after immigration, Hispanic participants treat formula as a backup plan, reflecting a rational adaptation for participants in the U.S. context and culture, where formula is viewed as a safe, endorsed, and readily available alternative, contrasting with perceptions in their home countries where formula might be seen as less accessible or even harmful. Hispanic participants’ access to health education and nutritious foods in WIC may also influence their decisions on infant feeding. A systematic review by Bigman et al. (2018) highlights the complex relationship between acculturation and breastfeeding behaviors among Hispanic women. This review revealed that higher levels of acculturation were inversely related to breastfeeding rates, regardless of income level [29]. Similarly, research conducted in rural Washington state with Hispanic women of low acculturation explored their perceptions, experiences, and attitudes toward breastfeeding. That study also found that despite a strong cultural and familial expectation to breastfeed, women faced challenges and conflicts adapting to life in the U.S., including embarrassment about breastfeeding publicly and economic pressures to return to work [30]. Our findings align with the literature where pregnant women have anticipated using formula as a backup in anticipation of resuming work. These findings underscore the need for anticipatory guidance about how to breastfeed through these transitions, accompanied by continued support, family-level interventions, and workplace policies that facilitate breastfeeding among Hispanic populations.

The findings of this study highlight a critical gap in Hispanic WIC participants’ breastfeeding knowledge and perception of WIC breastfeeding support. Although participants in our study shared a deep understanding of the benefits associated with breastfeeding, e.g., breastmilk being the most nutritious food for infants, a lack of awareness was observed regarding breastfeeding support resources to address common breastfeeding challenges, such as pain, low milk supply, infant satiety, and latching [2,3,31]. This disconnect leads Hispanic WIC participants to use the formula provided freely by the WIC program as a backup when faced with any breastfeeding challenge. The early introduction of formula is common in Hispanic culture, which might make Hispanic participants more comfortable than other ethnic groups with introducing formula to their babies when facing breastfeeding challenges. Thus, more anticipatory and culture-sensitive guidance and accessible skilled breastfeeding support within the WIC program are needed. The current study’s results align with previous research indicating that adequate breastfeeding support needs to include practical strategies for overcoming common breastfeeding obstacles [5,6]. Without such early and ongoing skilled lactation support, beliefs about the potential benefits of breastfeeding are unlikely to be converted into actual breastfeeding behaviors for many Spanish-speaking WIC participants.

While participants discussed using formula as an alternative backup plan if breastfeeding was not successful, no participants described other alternatives, such as lactation consultants, breast pumps, or donor milk. This omission signifies a knowledge gap but also highlights potential barriers to accessing these resources, whether due to linguistic challenges, cultural norms, or systemic hospital or WIC clinic barriers unique to Hispanic WIC participants. Moreover, rather than working with WIC to identify strategies to overcome anticipated hurdles with the help of WIC-provided support and resources, participants perceive WIC formula distribution to be an endorsement of the use of the formula. This shows a failure of the program to educate participants to navigate the initial breastfeeding challenges successfully and appropriately. This formula “backup” mindset may adversely affect the WIC breastfeeding promotion efforts and potentially reduce breastfeeding initiation rates. Previous studies have also highlighted the pivotal role of early and consistent support from WIC in achieving successful breastfeeding outcomes [7,8]. This study also suggests a complex interplay between cultural beliefs, the influence of social networks, and the perceived reliability of formula feeding, which collectively shape participants’ attitudes toward breastfeeding and formula use. This finding aligns with research indicating that social and cultural factors significantly impact breastfeeding intentions and practices among diverse populations [10,11,32]. 

Although participants described WIC communications as culturally and linguistically concordant and said they felt comfortable and supported in their relationships with WIC staff, there is an opportunity to build upon these strengths to ensure WIC is appropriately educating participants about how to use WIC support to address and overcome many early breastfeeding challenges without turning first to formula. This would include describing the WIC resources available, such as breastmilk pumps, peer support, lactation consultants, and ensuring healthcare providers are aware of these early intervention services. 

To address these challenges effectively, it is also crucial for the WIC program to enhance the anticipatory guidance provided to WIC participants during pregnancy, encouraging them to utilize Baby-Friendly Hospitals (BFHs) or engage with their healthcare team to understand the evidence-based maternity care practices that support breastfeeding. This approach is supported by evidence suggesting that individuals giving birth in BFHs exhibit higher exclusive breastfeeding rates, which can also contribute to closing some racial/ethnic breastfeeding disparities [33]. Overall, providing comprehensive, culturally sensitive, and linguistically appropriate breastfeeding education and resources could allow Spanish-speaking WIC participants to navigate breastfeeding challenges more effectively, thereby extending the duration of breastfeeding among this population.

This study has several limitations. Due to its exploratory nature, the study included a small sample size of 18 participants, limiting the findings’ generalizability. The study recruited participants from WIC clinics in Nevada, which may not accurately represent the diversity of experiences and perceptions among Hispanic participants in WIC nationally. Another limitation is the reliance on self-reported data, which may be influenced by recall bias or social desirability bias, especially regarding sensitive topics like breastfeeding practices and decisions. Furthermore, these findings are specific to the context of the WIC program and may not apply to other populations or settings with different breastfeeding support and recommendations.

## 5. Conclusions

This qualitative study provides important insights into the complexities surrounding breastfeeding perceptions, decisions, and practices among Hispanic WIC participants. While these participants were aware of the benefits of breastfeeding and often preferred to breastfeed, they viewed formula as an essential alternative due to the challenges associated with breastfeeding, such as pain, low milk supply, and the need to return to work. Hispanic culture strongly emphasizes breastfeeding, but there has been a shift toward formula feeding as the group has migrated to the U.S., often due to easy access to infant formulas, their perceived convenience, and recommendations by several health organizations. 

Most participants believed that WIC recommends breastfeeding and appreciated the culturally appropriate education and materials provided by bilingual WIC staff or through language lines. However, this study highlighted a crucial gap in breastfeeding knowledge and awareness about the available breastfeeding support via the WIC program. WIC can further enhance culturally and linguistically tailored breastfeeding support to meet the needs of the participants, with a significant focus on practical strategies to overcome initial breastfeeding challenges such as pain, low milk supply, etc., and on continuing breastfeeding through normative transitions such as returning to work to achieve successful breastfeeding outcomes. This result also provides evidence for further studies focusing on the complex nature of pregnant and postpartum people’s decision-making processes regarding infant feeding practices.

## Figures and Tables

**Table 1:** Characteristics of WIC participants interviewed (*n* = 18).

Response	Answer	Count (Total 18)	Percentage (%)
**Pregnancy Status**	Yes	7	39
	No	11	61
**Age**	18–24	8	44
	25–29	7	39
	30–34	2	11
	35–39	1	6
**Marital Status**	Married	5	28
	Unmarried, living with a partner	4	22
	Single (never married)	7	39
	Divorced, separated, or widowed	2	11
**Education Level**	Less than high school	2	11
	Graduate or high school or GED	7	39
	Some college but no degree	4	22
	Associate degree	2	11
	Bachelor’s degree or higher	3	17
**Employment**	Full-time work	2	11
	Part-time work	3	17
	Housewife	7	39
	Other	6	33
**Health Insurance**	None	4	22
	Medicaid	12	67
	Private	2	11
**Language Spoken at Home**	English Only	3	17
	Spanish Only	11	61
	Both	4	22
**Urban/Rural Residence**	Urban	16	89
	Rural	2	11
**Residence Years in USA**	Less than one year	2	11
	1–5 years	8	44
	5–10 years	3	17
	More than 10 years	5	28

**Table 2:** (**a**) Infant feeding plan of Hispanic pregnant WIC participants. (**b**) Infant feeding plan of Hispanic postpartum WIC participants.

(**a**)							
**Participant ID**	**Country of Origin**	**Gestational Weeks**	**Anticipated BF Months**	**Alternative IFP**	**Reasons for Change in IFP**		
P68	Nicaragua	12	12	BF	Not aware of alternative feeding practices but wants to only breastfeed for a year because it has all the nutrition that is needed for the infant’s growth.		
P4	Cuba	14	4	F	Breastfeed only for 3–4 months and start on solids such as “taro” or chicken meat to build baby’s stomach. Change to formula only in case there is no milk supply.		
P100	El Salvador	15	3–4	F	Formula feeding if there is low or no milk supply but, if possible, the participant wants to continue mixed feeding practice with 70% breastmilk and 30% of the formula in given situation.		
P26	Mexico	20	8–12	F	Due to job-related complications, formula would be a better option. BF will be very difficult with pumping and extracting breastmilk at work.		
P26	Mexico	20	8–12	F	Due to job-related complications, formula would be a better option. BF will be very difficult with pumping and extracting breastmilk at work.		
P31	Not Reported	21	8	F	Change to formula because it is difficult to produce breast milk in the first few days.		
P40	Cuba	28	6–12	F	Change to formula if there is a medical condition or low milk supply.		
(**b**)							
**Participant ID**	**Country of Origin**	**Postpartum Months**	**IFP at the Prenatal Stage**	IFP* in Hospital	IFP* after Discharge	IFP* at the Time of the Interview	Reasons for Change in IFP*
P88	Not Reported	2 Weeks	BF	BF	BF	BF	No change in IFP*; BF is easy because formula feeding requires bottle washing and cleaning.
P90	Not Reported	1	BF	BF	BF	BF	No change in IFP*; BF makes me feel happy.
P57	El Salvador	1	BF	MF	MF	MF	Changed to formula because baby would not latch on in the first few days.
P7	Unknown	1	BF	MF	MF	MF	Changed to formula at the hospital because of high bilirubin levels and then to MF because of low milk supply for 15 days.
P1	El Salvador	1.5	MF	MF	MF	F	BF was painful and uncomfortable; changed to formula after two days of no breastmilk production.
P53	Cuba	2	BF	MF	MF	F	Started with MF due to high bilirubin levels and low milk supply at the first few days; changed IFP* to exclusively for formula after infant rejected breastmilk.
P75	Colombia	2	BF	MF	MF	BF	Changed to MF only for 8 days due to low milk supply.
P56	Puerto Rico	3	BF	BF	BF	BF	No change in IFP/BF makes me feel super good.
P45	Colombia	5	BF	MF	MF	MF (BF + porridge)	Changed to mixed feeding because breastmilk is not enough, and infant does not gain satisfaction. Along with breastmilk, infant is receiving pumpkin porridge.
P76	Peru	7 ^1^	BF	BF (Pumping)	BF (Pumping)	MF	Changed to MF after 5 months of exclusive BF. Due to participant’s medical condition and infant’s high bilirubin, breastmilk was pumped.
P89	Mexico	7 ^1^	Not Sure	MF	MF	MF	Changed to MF because of low milk supply.

BF: breastfeeding; F: formula; MF: mixed feeding; IFP: infant feeding plan; IFP*: infant feeding practice. ^1^ When participants completed the screening surveys, they were still six months after delivery.

## Data Availability

The data presented in this study are not available from the corresponding author due to the sensitive nature of the interview scripts.
